# Evidence-informed methodological approaches for coupled neuromusculoskeletal-finite element analysis workflows in orthopaedic biomechanics

**DOI:** 10.3389/fbioe.2026.1875782

**Published:** 2026-09-03

**Authors:** Alireza Y. Bavil, Ayda Karimi Dastgerdi, Rod S. Barrett, Bradley M. Cornish, Matthew J. Hambly, Matthew Carrall-Worsey, Claudio Pizzolato, Amir Esrafilian, Dale Robinson, Laurent Frossard, David John Saxby, Stefanie Feih, Christopher P. Carty, David G. Lloyd

**Affiliations:** 1 School of Mechanical Engineering and Industrial Design, Griffith University, Gold Coast, QLD, Australia; 2 Australian Centre for Precision Health and Technology (PRECISE), Griffith University, Gold Coast, QLD, Australia; 3 School of Allied Health, Sport and Social Work, Griffith University, Gold Coast, QLD, Australia; 4 Department of Technical Physics, Faculty of Science, Forestry and Technology, University of Eastern Finland, Kuopio, Finland; 5 School of Human Sciences, University of Western Australia, Perth, WA, Australia; 6 School of Mechanical and Mining Engineering, University of Queensland, Brisbane, QLD, Australia; 7 School of Exercise and Nutrition Sciences, Queensland University of Technology, Brisbane, QLD, Australia; 8 School of Medicine and Dentistry, Griffith University, Southport, QLD, Australia

**Keywords:** digital twin, finite element analysis, neuromusculoskeletal modelling, orthopaedic biomechanics, surgical planning and simulation

## Abstract

Orthopaedic surgical outcomes remain variable due to the poorly characterised, patient-specific post-surgical mechanical and physiological environment of implants and tissues. Differences in anatomy, alignment, neuromuscular function, and movement govern surgical-site loading and the tissue-level environment that drive healing. Coupled neuromusculoskeletal-finite element analysis (NMSK-FEA) workflows link these factors to the tissue-level mechanical and physiological environment, enabling preoperative evaluation of surgical strategies. This paper outlines current methodological considerations for the development and application of coupled NMSK-FEA workflows in orthopaedic biomechanics and identifies key priorities for their translation into clinically useful decision-support tools. We discuss methodological considerations and rationale for creating personalised MSK and NMSK models, patient-specific FEA geometry and material definition, transfer of NMSK outputs into FEA motion, loading, and boundary conditions, and selection of clinically relevant output measures. The workflow enforces NMSK and FEA parameter and boundary condition constraints to generate physiologically plausible outputs. Case studies of anterior cruciate ligament reconstruction and proximal femoral osteotomy are presented as methodological exemplars to illustrate how these NMSK-FEA workflow recommendations can be operationalised. These examples of NMSK-FEA workflows used in clinical cases show that mechanically optimal solutions can differ substantially between patients, thereby reinforcing the need for personalised biomechanical assessment. This paper presents practical recommendations for standardising and validating workflows and communicating key findings in a format that supports clinical decision making, laying the groundwork for prospective clinical validation. Together, these recommendations help move orthopaedic surgery planning beyond population averages towards patient-specific decision support at the point of care.

## 1 Introduction

Musculoskeletal (MSK) conditions are a major cause of pain, disability, and healthcare burden worldwide (Gill et al., 2023), and orthopaedic interventions are required to restore function, relieve symptoms, and delay structural deterioration. Yet outcomes after orthopaedic procedures such as arthroplasty, osteotomy, ligament reconstruction and fracture fixation remain highly variable, and revision surgery remains common across indications and patient populations (Feng et al., 2026; Guarino et al., 2023; Lewis et al., 2022; Liukkonen et al., 2022). In current practice, orthopaedic surgical decisions are commonly informed by clinical examination, medical imaging, alignment measurements, implant templating, and population-level outcomes data (Tanzer and Makhdom, 2016). In selected settings, these steps may be supplemented by three-dimensional planning or patient-specific instrumentation, but these tools often remain centred on anatomy, alignment, and implant positioning (Alemayehu et al., 2021). Consequently, conventional workflows have limited capacity to anticipate how patient-specific neuromuscular control and functional loading interact with the planned correction, fixation, or implant after surgery. Outcome variability therefore reflects, in part, the strong influence of patient-specific anatomy, alignment, soft-tissue behaviour, and neuromusculoskeletal (NMSK) control of movement on postoperative mechanics, including joint kinematics, contact loading, tissue stress, and the local mechanical environment for fixation and healing. Crucially, the contribution of NMSK control reflects more than structural differences alone: these mechanical responses are shaped not only by MSK structure, but also by how the nervous system organises and coordinates muscle activation, joint stabilisation, and tissue loading during movement through the anticipatory nature of neural control. In individuals with altered or pathological neuromuscular control, the neural control and resulting subject-specific loading patterns may differ substantially from typical assumptions and can contribute to abnormal tissue loading, deformity progression, and variable surgical response. However, in most cases, anatomy, movement, loading, and tissue mechanics are assessed in an unconnected fashion, and with limited consideration of how these are affected by the neural control strategies. This limits the capacity to predict how an intervention will alter these mechanically relevant outputs in an individual patient and, in turn, influence their surgical outcome.

These challenges highlight a limitation of conventional one-size-fits-all planning approaches, which do not explicitly account for patient-specific differences in anatomy, alignment, neuromuscular control, and physiological loading. As these factors influence the mechanical environment at the surgical site, there is increasing motivation to develop surgeon-facing preoperative planning tools that can predict the mechanical consequences of a planned intervention on a subject-specific basis (Liu et al., 2025). Coupled NMSK-FEA modelling provides one means of addressing this need (Dastgerdi et al., 2025a). In this framework, NMSK models estimate subject-specific physiological inputs, such as joint kinematics, muscle forces, and joint contact loads, which can then be applied as boundary conditions in FE analyses. This enables prediction of tissue and implant stresses, strains, and contact mechanics under anatomically, physiologically, and functionally realistic conditions. The key value of the combined approach lies not only in representing patient-specific anatomy, but in linking subject-specific movement and neuromuscular function to the tissue-level mechanical environment that a surgical intervention imposes. This is particularly relevant in orthopaedics, where aberrant muscle activation, co-contraction, or impaired coordination may substantially alter joint loading (Uhlrich et al., 2022), soft-tissue mechanics, and healing conditions, yet are rarely captured in standard planning workflows. By integrating neural and musculoskeletal function with FEA, coupled NMSK-FEA frameworks offer a pathway to predict how structure and control interact to shape tissue-level loading before intervention is performed.

The workflow presented is grounded in established engineering principles. Engineering adopts a systematic design approach that deconstructs complex systems into components, which are further decomposed into elements that are based on physical and physiological fundamentals. These components are analysed through ever-increasing use of validated computational models, supported by experimentation, and then recombined to create novel solutions, such as the surgical procedures and implants presented here. The static and dynamic behaviour of these computational models is governed by their underlying mathematical formulations and discretisation. Examples include differential equations describing muscle activation and motion, and the number and type of finite elements used to represent bone. Model behaviour is further shaped by parameters such as optimal fibre length in Hill-type muscle models and the continuum material properties assigned to cortical and trabecular bone. The use of computational modelling and simulation, or digital twins, as exemplified in Industry 4.0 in the digitalisation of industry and extension to Industry 5.0 with co-operation between human and machine (Saxby et al., 2023), is gaining increasing recognition and acceptance by medical regulatory bodies such as the European Medicines Agency (Pappalardo et al., 2022; Karanasiou et al., 2025) and the U.S. Food and Drug Administration (Morrison et al., 2017). Formal guidance for verification and validation of computational modelling and simulation has been provided by the American Society of Mechanical Engineers (ASME, 2018), and this guidance has recently been applied to orthopaedic implants (ASME, 2026). Furthermore, recent work on clinically oriented workflows increasingly frames this paradigm as a feasible pathway towards patient-specific orthopaedic decision support (Andres et al., 2025).

This study outlines methodological recommendations for computational modelling and simulation in the development and application of coupled NMSK-FEA workflows for orthopaedic biomechanics with a primary focus on preoperative planning and comparison of candidate surgical strategies. This evidence-informed perspective translates specialised workflow experience, published evidence, and established modelling principles into actionable best-practice recommendations for coupled NMSK-FEA modelling. We demonstrate these recommended methodological considerations through two in-depth case studies and identify key methodological priorities for advancing these frameworks towards robust, scalable, and clinically implementable patient-specific decision-support tools for a wider range of orthopaedic interventions and healthcare settings.

## 2 Musculoskeletal modelling

An MSK model is a mathematical representation of the human musculoskeletal system that is used to analyse movement and estimate the associated internal tissue forces that cause movement, which cannot be directly measured *in vivo*. MSK models incorporate skeletal, joint, and muscle-tendon anatomy and use multi-body dynamics to simulate or predict motion, enabling estimation of kinematics and forces, at the level of the whole body, joints, and/or individual tissues (Delp et al., 2007). MSK modelling is widely used to investigate disease mechanisms, understand injury, inform surgical and conservative interventions, assess performance, and support the design of prostheses, orthoses, and assistive devices (Bulat et al., 2019). Best practice guidelines for MSK modelling have previously been reported (Hicks et al., 2015) and so only a brief overview of current practice is provided here.

Research has demonstrated that subject-specific representation of anatomy, joint mechanics, and muscle-tendon geometry can substantially improve the physiological and anatomical plausibility of musculoskeletal models (Kainz et al., 2021). However, structural personalisation alone does not determine how movement is generated in an individual. Muscle forces and joint loading also depend on how the nervous system coordinates muscle activation to generate movement while satisfying other control objectives. Consequently, anatomy-based personalisation alone is often insufficient for predicting subject-specific MSK loading patterns relevant to orthopaedic decision-making. For this reason, Section 2.2 focuses on modelling approaches that extend conventional MSK frameworks by incorporating neural control, including physiological muscle activation, in conjunction with structural personalisation.

### 2.1 Patient-specific MSK models

MSK models can be constructed with varying levels of subject specificity. Generic models are typically derived from single or multiple cadaveric measurements or medical imaging of living subjects to define segment geometry, joint kinematics, muscle-tendon paths, and associated properties such as fibre length, pennation, and strength (Carman et al., 2022; Rajagopal et al., 2016). The simplest level of personalisation involves linearly scaling a generic model to estimate subject dimensions and inertial properties. Commonly, this is done by computing the ratio between pairs of markers placed on bony landmarks in experimental space and those corresponding landmarks in digital model space (Lund et al., 2015; Winby et al., 2008). Scaling factors, also known as ratios, can be computed isotropically for an entire segment using one or an average of its measures. Alternatively, they can be applied in an anisotropic fashion to each dimension of the segment (e.g., length, width, depth). Such scaling is computationally efficient and supported by conventional computational modelling software such as OpenSim or AnyBody.

However, linear scaling does not capture subject-specific variations in bone morphology, joint alignment, or muscle-tendon architecture (Klemt et al., 2019). At the other end of the spectrum, highly personalised models can be generated from medical imaging, where bones are segmented, joint definitions are derived from morphology, and muscle attachments, pathways, and strength are informed by muscle volume (Fukuda et al., 2017; Modenese and Renault, 2021; Hainisch et al., 2012). Despite their improved fidelity, such image-based subject-specific modelling approaches remain time-consuming to construct and execute and are not yet practical for routine orthopaedic applications. In coupled NMSK-FEA workflows, this creates a practical anatomical-consistency challenge when CT- or MRI-derived geometries are used to generate the FE model, while the corresponding MSK model remains linearly scaled or only partially personalised. Ideally, the same imaging-derived bone morphology should inform both modelling environments, so that skeletal geometry, joint definitions, muscle-tendon paths, and force directions remain anatomically consistent throughout the coupled workflow. This requirement is particularly important in severe musculoskeletal deformities, where abnormal morphology exceeds the assumptions of linear scaling and requires additional model adaptation. Intermediate personalisation strategies are therefore commonly adopted to balance subject specificity with computational and practical feasibility (Hicks et al., 2015). These intermediate approaches, rather than using simple linear scaling to segment lengths (Harrington et al., 2007; Hara et al., 2016), can apply statistical shape modelling to large datasets that account for the principal directions (vectors) of variation in human morphometry (De Roeck et al., 2021). These approaches can use limited inputs, such as 3D motion-capture estimates of anatomical landmarks, to adapt skeletal morphometry and muscle attachment locations, estimate joint centres (Yeung et al., 2020; Zhang et al., 2016), calculate muscle-tendon-unit pathways, tune muscle-tendon parameters (e.g., optimal fibre length and tendon slack length) to ensure physiological plausibility (Modenese et al., 2016), and scale muscle strength based on anthropometrics or imaging-derived estimates (Handsfield et al., 2014).

Once the model and its internal properties are defined and determined to match the subject of the study sufficiently well, the next step is to mobilise the model. Segmental and joint motions for a given MSK model are typically estimated using inverse kinematics analysis, where the segmental and joint model constrains the estimated kinematics to be anatomically plausible. In this, a weighted least-squares optimisation minimises differences between paired experimental and model 3D marker trajectories over time (Delp et al., 2007). Generalised joint loads (e.g., forces and moments) are then calculated using inverse dynamics, solving Newton-Euler equations of motion using the ground reaction forces and moments applied to the foot segment or other bodies in contact with external reaction objects (Seth et al., 2018). At this level, a clinically implementable MSK workflow minimally requires subject anthropometrics, task-specific kinematic data, and external reaction loads sufficient to estimate joint motion and net joint kinetics. These inputs may come from full motion capture, validated wearable-sensor or clinical gait protocols, or population-informed assumptions, with decreasing subject specificity as measurement detail is reduced. Collectively, these steps are termed inverse approaches to MSK modelling. Although this sequential inverse approach is computationally efficient, it introduces dynamic inconsistencies due to modelling assumptions and measurement errors (e.g., scaling inaccuracies, inertial properties, joint constraints, and marker movement artefact) (Hatze, 2002; Riemer et al., 2008). Alternative formulations address some of these limitations by solving model motions and dynamics concurrently, for example, through forward simulation (Thelen and Anderson, 2006), tracking-based approaches (Lin and Pandy, 2017), or concurrent optimisation-based MSK modelling (Smith et al., 2019) of muscle activations and joint kinematics, albeit at substantially higher computational cost. Whilst these approaches can account for dynamic inconsistencies between motion, muscle forces, and joint moments, they do not account for an individual’s muscle activations and resulting actions that generate the computed joint moments. Indeed, the actions of muscle can vary greatly between people (Schmitt and Rudolph, 2007; Hug et al., 2019), and within people across different tasks and control criteria (Osu et al., 2002; Buchanan and Lloyd, 1995; van der Gon et al., 1991; Tax et al., 1990; Tax et al., 1989).

More fundamentally, even highly personalised MSK models can only represent neural control indirectly when muscle recruitment is inferred from instantaneous mechanical objectives. This is common in static optimisation approaches that estimate muscle activation and forces to match net joint moments (Princelle et al., 2025). Yet muscle force at any instant reflects the prior history of excitation and force production as muscles serve multiple functions that extend beyond generating net joint moments. Muscle activation and action are coordinated across time to satisfy multiple control objectives, including accommodating fatigue (Refai et al., 2024), managing multi-joint and multi-directional force transmission (Sartori et al., 2012a), regulating joint impedance to counter movement perturbations (Cop et al., 2024; Cop et al., 2022; De Serres and Milner, 1991), stabilising joint secondary kinematics (Besier et al., 2003), attaining appropriate regional loading of joint articular surfaces (Kian et al., 2019), producing energetically efficient yet powerful movement (Worsey et al., 2025), and/or compensating for pathology (Veerkamp et al., 2024; Davico et al., 2022). These recruitment strategies have proven to be subject-specific and cannot be fully determined from anatomy and/or joint kinematics and kinetics alone. Thus, when the clinical questions depend on subject-specific muscle activation and loading, structural personalisation of an MSK model is necessary but insufficient, motivating extension to patient-specific neuromusculoskeletal models. Nevertheless, EMG-informed modelling depends on the availability and quality of EMG data, which may not always be feasible to collect in all clinical or research settings. When EMG recordings are limited or incomplete, EMG-assisted, EMG-hybrid, and synergy-informed approaches can reduce reliance on large EMG datasets by using measured excitations with optimisation or low-dimensional synergy constraints to estimate unmeasured muscle activity (Hambly et al., 2025a). Where EMG data are unavailable or unreliable, or where their collection is clinically impractical, the broader modelling workflow can still be applied, but the assumptions and limitations of the selected muscle and joint force estimation approach should be explicitly acknowledged when interpreting downstream FE outputs.

### 2.2 Patient-specific neuromusculoskeletal (NMSK) models

NMSK models extend MSK models by incorporating neural control, enabling physiologically plausible predictions of muscle activations and forces, with the resulting joint moments and contact forces (Zajac, 1989). These models typically represent activation dynamics (Lloyd and Besier, 2003; Manal and Buchanan, 2003; Winters, 1995; Manal and Buchanan, 2013), contraction dynamics using a Hill-type muscle model (Hill, 1938), and now joint mechanics (Esrafilian et al., 2021). Together, these components aim to capture key physiological features of muscle behaviour, including activation-dependent force generation, electromechanical delay, and the dependence of muscle output on muscle-tendon state and contraction history. The behaviour of each muscle-tendon unit is governed by non-linear force-length-velocity functions that are scaled using subject-specific muscle-tendon parameters (e.g., optimal fibre length, tendon slack length, maximum isometric force) (Pizzolato et al., 2015; Kian et al., 2021; Hoang et al., 2018; Romanato et al., 2023). Since these parameters are influenced by anatomy, training history, and pathology, their personalisation is important when estimating subject-specific muscle forces and joint loading. However, many of these critical model parameters cannot feasibly be measured either because the methods are invasive for use in humans, or because their measurement protocols would be prohibitive in terms of time. In practice, available measurements provide partial constraints rather than complete parameter definition: MRI can inform muscle volume and cross-sectional area, ultrasound can provide targeted estimates of fascicle length, and dynamometry can constrain force-generating capacity. As an alternative to direct measurements, computational methods such as model calibration are used prior to model execution, all with suitable known physiological constraints. Accordingly, expected accuracy is not fixed for a given muscle-tendon parameter or imaging modality, but depends on the quality of the available measurements and the methods used to estimate and calibrate model parameters. The measurement or estimation source, parameter bounds, and physiological plausibility checks should therefore be reported when personalised muscle-tendon properties are used.

Model calibration is achieved by iteratively adjusting these muscle-tendon parameters to minimise the difference between inverse dynamics and model-predicted joint moments, often alongside additional physiological objectives (Hambly et al., 2025a; Lloyd and Besier, 2003; Pizzolato et al., 2015; Meinders et al., 2022). Beyond improving moment tracking, calibration enhances physiological plausibility by implementing constraints of model parameters that constrain muscle behaviour, for example, by ensuring fibres operate within appropriate regions of the force-length relationship (Pizzolato et al., 2015), incorporating multiple trials or tasks (Lloyd and Besier, 2003; Kian et al., 2021), and using multi-degree-of-freedom models with biarticular muscles (Sartori et al., 2012a). Calibration is therefore not only a numerical fitting step but also a process of implementing constraints that tailor the physiological and anatomical muscle model to the individual. This is particularly relevant in pathological conditions, where persistent abnormal activation may alter muscle and tendon properties and where small geometric differences can have disproportionately large effects on muscle mechanics and joint loading (Davico et al., 2022; Rabbi et al., 2024; Davico et al., 2020).

Historically, calibration has been a major barrier to clinical translation due to high computational cost, with optimisation methods such as simulated annealing or particle swarm often requiring hours to converge (Refai et al., 2024; Corana et al., 1987; Schutte et al., 2005). Recent developments have significantly improved efficiency by reformulating NMSK models within auto-differentiable frameworks, enabling gradient-based optimisation and reducing calibration time from hours to minutes, while facilitating integration with neural network approaches and muscle synergy-informed excitation synthesis (Hambly et al., 2025a). Emerging methods also optimise muscle pathways (Ao et al., 2023) or joint mechanisms to estimate secondary knee-joint kinematics during calibration, improving anatomical and dynamic consistency while reducing modelling errors. Once calibrated, the model can be executed to estimate muscle and joint mechanics.

NMSK model execution uses joint kinematics and muscle excitations as primary inputs. Joint angles obtained from inverse kinematics are used to compute muscle-tendon unit (MTU) kinematics, including lengths and moment arms, typically defined by muscle paths and wrapping surfaces in the underlying MSK model. While improved personalisation of muscle attachments and pathways can reduce discontinuities and errors (Ao et al., 2023), this computation of MTU kinematics can be demanding. To address this, surrogate formulations such as polynomial functions (Menegaldo et al., 2004), multidimensional B-splines (Sartori et al., 2012b), and neural network approximations (Cornish et al., 2024) have been developed to provide smooth, differentiable mappings from joint angles to MTU lengths, ensuring continuity when computing moment arms via differentiation.

The number of muscles acting about a joint exceeds the available mechanical degrees of freedom, giving rise to the well-known problem of muscle redundancy. When muscle excitations are not directly measured by EMG, muscle activations and their force contributions to joint moments are commonly estimated using optimisation-based approaches, including static and dynamic optimisation (Crowninshield et al., 1978; Patriarco et al., 1981) and computed muscle control (Thelen and Anderson, 2006; Thelen et al., 2003). These methods infer muscle recruitment primarily from a limited set of mechanical cost functions, with limited representation of prior muscle history or anticipated future demands associated with anticipatory (predictive) motor control of human movement (Besier et al., 2003; Dhaher et al., 2005; Dhaher et al., 2003; Buchanan et al., 1996). Consequently, purely mechanics-based estimates may fail to capture the time-varying muscle coordination strategies discussed above, which serve multiple control objectives and are shaped by an individual’s history, training, and pathology. Although these objectives must remain mechanically and dynamically consistent, they are embedded within subject-specific muscle coordination strategies. As such, optimisation based solely on mechanical objectives may not accurately reproduce subject-specific muscle activation patterns and subsequent muscle, joint contact, and tissue forces (Princelle et al., 2025; Kian et al., 2019; Davico et al., 2022; Rabbi et al., 2024; Davico et al., 2020; Crossley et al., 2025). Using EMG is a practical and pragmatic way to reveal the individual’s coordinated muscle activation patterns.

Calibrated EMG-informed methods address many of these limitations by incorporating experimentally measured neural activity (EMG) to generate subject- and task-specific muscle force predictions. The EMG can be used directly (EMG-driven) (Lloyd and Besier, 2003), combined with optimisation to estimate excitations for unmeasured muscles (EMG-hybrid) (Sartori et al., 2014), or used to constrain and adjust excitation patterns while ensuring consistency with joint moments (EMG-assisted) (Pizzolato et al., 2015). Because EMG provides direct insight into the individual’s neural control strategy, these approaches are particularly valuable when activation patterns are atypical, such as in orthopaedic conditions associated with neurological conditions (e.g., cerebral palsy, stroke, spinal cord injury).

Building on Bernstein’s seminal work (Bernstein, 1967), decades of research have shown that muscles are not controlled independently but are activated in coordinated spatial and temporal synergies (Hambly et al., 2025a; Rabbi et al., 2022; Walter et al., 2014; Neptune et al., 2009). Muscle synergies reduce redundancy and simplify motor control by representing EMG-derived muscle excitations in a reduced-dimensional space. These synergies, therefore, provide a physiologically consistent constraint for calibrating and executing NMSK, enabling reliable prediction of muscle activation and forces in both typical and pathological movement (Rabbi et al., 2024; Walter et al., 2014; Sartori et al., 2013; Sartori et al., 2015). Although further work is required to optimise synergies and synthesise unmeasured excitations during execution, recent improvements in computational efficiency of the synergy-informed approaches enable integration of calibration and execution, opening the possibility for near real-time model updating and adaptation to physiological changes due to, for example, fatigue (Hambly et al., 2025b).

All these EMG-informed NMSK approaches incorporate the aforementioned physiological constraints and parameters that shape muscle activation and contraction dynamics in calibration and execution. Therefore, they result in models that generate subject-specific predictions of experimental muscle activity and, importantly, joint (knee, hip, shoulder) and tissue (Achilles tendon) loading across many different tasks, such as walking, running, cycling and various training routines (Princelle et al., 2025; Kian et al., 2019; Hoang et al., 2018; Crossley et al., 2025; Bennett et al., 2022; Devaprakash et al., 2022). While direct validation of individual muscle forces remains challenging because they are rarely measurable *in vivo*, their physiological plausibility can be assessed indirectly through agreement with EMG patterns, joint moments, tendon loading, and joint contact forces (see Table 1).

**TABLE 1:** Summary of key practical considerations and workflow recommendations for NMSK modelling to facilitate subject-specific NMSK-FEA workflows.

Category	Simulation variables and processes	Key dependencies	Methodological recommendation
Input signals	Experimental muscle excitations (e.g., surface, indwelling, or high-density electromyograms)	Setup/configuration of electrodes (e.g., size, materials, positioning, orientation, skin preparation, electrotechnical noise); normalisation reference (maximal isometric voluntary contractions, sub-maximal standard contractions, or dynamic)	Use standardised EMG processing; normalise to maximal voluntary contractions or high-intensity task-relevant reference; verify timing alignment with kinematics and kinetics
	MTU kinematics (i.e., instantaneous MTU lengths, moment arms, and lines of action)	Kinematics; musculoskeletal geometry; model scaling; joint definitions	Use subject-specific scaling and personalisation where possible; verify moment arms and MTU lengths against literature or imaging; ensure kinematic consistency with no discontinuities
	Target joint moments **(**predicted values compared to inverse dynamics or other experimental values**)**	Kinematics; external forces/moments; musculoskeletal model geometry, joint definitions, inertial parameters	Derived from measures with high confidence (e.g., inverse dynamics, dynamometer); filter at common or lower frequency to MTU kinematics and muscle excitations (controller signal must be of equal or greater frequency content than the output signal); moments from open-chain exercises may require personalised inertial parameters
NMSK model calibration	Uncalibrated NMSK model generation	Musculoskeletal model; model scaling; model personalisation (e.g., bone geometry, joint definitions, MTU paths and strength); model optimisation (e.g., MTU variables)	Personalise musculoskeletal model geometry and MTU paths where possible (e.g., from MRI/CT); perform initial tuning and scaling of tendon slack length and optimal fibre length using established dimensionless operating curves across multiple degrees of freedom (e.g., joints); estimate maximum isometric force from medical imaging or from known relationships to anthropometrics
	Neuromusculoskeletal model calibration	Calibration procedure; experimental data (e.g., MTU kinematics, experimental excitations, and joint moments); parameter bounds; degrees of freedom considered, loss functions	Perform subject-specific calibration; set tuneable parameters bounds to physiologically plausible ranges; apply penalties to restrict non-physiological fibre operating ranges (e.g., at extremes of lengths) and reduce total muscle force; verify the calibrated model can accurately predict joint moments and the normalised fibre lengths operate in the nominal range; use synergy-informed calibration when some muscles do not have experimental excitations
NMSK model execution	Execution settings	Neural mode; optimisation weightings	Use EMG-assisted or EMG-hybrid when target joint moments are known; use CEINMS-Optimise to identify optimal excitation tracking weights
	Adjusted muscle excitations (from EMG-assisted execution)	Experimental muscle excitations; MTU lengths; moment arms; calibrated NMSK model; joint moments; objective weights	Report EMG tracking error; justify optimisation weights; assess the physiological plausibility of predicted muscle coordination patterns against task- and population-appropriate reference data, including activation timing/phasing and antagonist co-activation where relevant
​	Muscle activations	Muscle excitations (experimental values); calibrated NMSK model (predicted values)	Ensure physiological plausibility by verifying that predicted muscle activations remain within feasible bounds (0–1) and exhibit task- and population-appropriate timing, phasing, and coordination patterns, including antagonist co-activation where relevant. Where EMG data are available, confirm that predicted activation timing and overall patterns are reasonably consistent with the experimental signals. This practice can also be implemented in non-neural optimisations, such as static optimisation, to ensure the activation patterns are in reasonable agreement with experimental data
	Fibre lengths	Muscle activations; MTU lengths; calibrated NMSK model	Ensure fibre lengths operate within physiological ranges; verify against expected force-length behaviour for the specific task. For walking, typical values are commonly considered to be 0.8–1.2
	Tendon strain	Calibrated tendon slack length; Predicted tendon length (depends on MTU dynamics)	Use physiologically plausible elastic tendon model (e.g., no excessive strains such as those >10%, no bulk compression); compare to literature values where available
	Muscle forces	Muscle activations; MTU lengths; calibrated NMSK model	Verify large co-contraction against experimental muscle excitations. Muscle forces can be indirectly validated for physiological plausibility by performing subsequent joint reaction analysis and comparing to instrumented implants
	Joint moments	Muscle forces; moment arms	Validate against inverse dynamics or experimental measurements of joint moments and report tracking error; justify optimisation weights; ensure consistency across tasks
	Joint contact forces	Joint reaction analysis outputs	Validate against *in vivo* or literature data where available. Investigate abnormally excessive values as potential modelling or processing errors. Peak magnitude, temporal pattern, and expected unloading events, such as toe-off during gait, can be used as plausibility checks before rigorous validation

Although not derived directly from relevant industrial standards (as they may not exist for many workflow steps), these recommendations are structured to align with broader American Society of Mechanical Engineers (ASME) expectations for computational modelling and simulation, particularly with respect to context of use, transparent reporting of model assumptions and inputs, and credibility assessment through verification and validation (ASME, 2018; ASME, 2026). CEINMS, Calibrated EMG-Informed Neuromusculoskeletal Modelling Toolbox.

### 2.3 NMSK output measures

The patient-specific NMSK model outputs define the applied mechanical environment of tissues, implants, and reconstructed structures. These include muscle forces, joint contact forces, joint moments, and time-varying joint kinematics, and embody the effects of subject-specific neural control, muscle coordination, and physiological muscle behaviour, and therefore provide a more faithful representation of tissue loading than inputs derived from mechanical optimisation solutions alone. These quantities provide the loading conditions through which FE models can resolve internal tissue-level stresses, strains, and contact mechanics. Their selection should be guided by the requirements of the downstream FE application (Section 3.3), as not all outputs are relevant to every model. For example, resultant joint contact force may be sufficient in some applications, but decomposition to components may be necessary when the FE objective is to resolve localised mechanics within a joint. To ensure meaningful inputs for FE models, all transferred quantities must be represented using consistent anatomical definitions, coordinate systems, force directions, lines of action, and temporal alignment. A practical quality-control step is to verify that NMSK outputs remain dynamically consistent with the measured task and modelling assumptions before FEA transfer, rather than assuming that outputs from a calibrated pipeline are automatically transferable. More broadly, automated and standardised load-transfer workflows are likely to improve reproducibility and reduce user-dependent variability in coupled NMSK-FEA analyses.

## 3 Finite element analysis

FEA is an established computational framework that discretises complex geometries and solves governing field equations to quantify internal stresses, strains, and contact mechanics that are not directly measurable *in vivo* (Heller, 2022; Taylor and Prendergast, 2015). In orthopaedic biomechanics, FE models range from single-compartment models (e.g., modelling individual bones such as the femur or tibia) to multi-compartment models that incorporate several tissues with contact mechanisms, depending on the underlying mechanobiological question (Bavil et al., 2024; Erdemir et al., 2019; Karimi Dastgerdi et al., 2023). This distinction is particularly important when FEA is coupled with NMSK because the validity of FE predictions depends on the appropriateness of the quantities transferred from the NMSK model and how they are applied as FE loading and boundary conditions (Bavil et al., 2024). Joint-level models typically use joint reaction forces, moments, or kinematic constraints to reproduce articular contact mechanics (Karimi Dastgerdi et al., 2023), whereas organ-level models more often apply muscle and joint contact forces to investigate structural responses of individual bones and/or implants (Bavil et al., 2024). The following sections outline key methodological steps, considerations and evidence-informed modelling choices underlying NMSK-FEA in orthopaedic biomechanics (see Table 2 for summary).

**TABLE 2:** Summary of key practical considerations and workflow recommendations for transferring NMSK outputs into FE loading and boundary conditions in orthopaedic biomechanics.

Category	Modelling detail	Key dependencies	Methodological recommendation for joint-level FE models	Methodological recommendation for single-bone FE models
Model definition	Mechanical objective of the FE model	Orthopaedic question; tissue of interest; level of analysis	Define loading to reproduce realistic articular contact mechanics	Define loading to preserve physiological load transfer through the bone or bone-implant construct while preventing rigid body motion
Boundary conditions	Prescribed kinematics	Motion task; joint definition; fixed-body coordinate system	One side of the joint is commonly fixed and the other mobilised, with joint kinematics together with joint contact forces and moments (re)expressed in the coordinate system of the fixed body	Full joint kinematics are generally not the primary boundary condition; constraints should instead stabilise the model without distorting physiological load transfer. Inertia relief method can be used to avoid non-physiological fixation regions
	Initial mechanical state	Joint pose; contact state; ligament definition; pre-strain assumptions	Include initial joint pose, contact state, and ligament pre-strain as part of the boundary-condition setup because they can substantially influence predicted tissue mechanics	Ensure the spatial placement of the bone is consistent with the NMSK-derived loading state and the anatomical configuration of the FE model
Applied loads	NMSK quantities transferred to FE	Downstream FE objective; availability and reliability of NMSK outputs	Transfer joint contact/reaction forces, joint moments, forces of muscles spanning the target joint, and time-varying joint kinematics as primary FE inputs	Transfer muscle and joint forces as primary FE inputs; include resultant moments of missing forces where the segment is partially represented
	Form of joint loading	Joint centre definition; coordinate system; required force-moment relationship	Apply resultant joint forces and moments through a reference point representing the functional centre of rotation, coupled to the relevant body or cartilage surfaces to preserve the correct force-moment relationship	Represent joint loading through reference points associated with anatomically relevant joint regions, coupled to the bone surface to simulate the articular cartilage load experienced by the bone
	Representation of muscle loading	Muscle attachment locations; via points; line of action definitions; anatomical correspondence between NMSK and FE	Muscle effects may be represented implicitly through muscle-force-derived moments superimposed onto inverse-dynamics moments, or explicitly by applying forces of muscles spanning the joint along their lines of action	Muscle forces should be applied explicitly. Their lines of action should be derived from the attachment site and the next immediate via point in the NMSK model. They should be applied using reference point coupling to anatomically relevant muscle attachment surfaces on the bone
	Force directions and lines of action	Exported NMSK vectors; attachment geometry; coordinate transformation	Confirm that force directions and lines of action are consistent with the joint-level mechanical representation and intended contact mechanics	Confirm that muscle and joint force directions remain consistent after transformation into the target bone coordinate system
	Temporal representation of loading	Activity type; FE computational strategy; time resolution required	For routine activities, downsampling or selection of key loading events is often sufficient; quasi-static FE implementations are generally appropriate unless the question specifically requires transient analysis	Representative loading instants are often sufficient for routine activities when quasi-static structural response is the target
Constraints	Constraint strategy	Structural stability of FE model; number and location of constraints	Avoid artificial constraints on the articular tissues and preserve the intended contact formulation	Apply only minimal, anatomically justified constraints; consider an isostatic 3-2-1 strategy or inertia relief where appropriate; verify that constraints suppress rigid-body motion without creating artificial reaction forces, local stress concentrations, or distorted physiological deformation
	Surface coupling strategy	Surface region selection; anatomical fidelity; FE formulation	Couple the reference point to the relevant body or cartilage surfaces while avoiding artificial constraints on contact surfaces of articular tissues	Couple anatomically relevant surface regions to reference points representing joint centres or muscle attachment locations to distribute loads smoothly over the corresponding regions in the FE model
Model alignment	Coordinate system consistency	Anatomical definitions; model alignment; load export procedure	All transferred forces, moments, and kinematics should be expressed using consistent anatomical definitions and coordinate systems between the NMSK and FE models	Muscle and joint forces must first be expressed in the target bone coordinate system before application
	Anatomical correspondence between NMSK and FE	Joint centre location; muscle attachment regions; segmentation/morphology; registration	Ensure correspondence between the NMSK joint centre, FE joint reference point, articulating surfaces, and any ligament definitions	Ensure correspondence between NMSK muscle attachment and via-point definitions, and FE coupling regions or attachment surfaces
Verification and modelling risk	Verification after load transfer	Export/import procedure; equilibrium check; modelling assumptions	Verify transferred loading after import to confirm force and moment equilibrium and consistency with the prescribed task	Verify transferred loading after import to confirm force and moment equilibrium and that no spurious rigid-body behaviour or artificial reactions have been introduced
	Typical modelling risk if poorly specified	Combination of all above dependencies	Incorrect joint centre definition, inconsistent coordinate systems, or artificial constraints may lead to non-physiological contact mechanics	Incorrect force transformation, poorly defined muscle line of action, or over- or under-constraining the FE model may lead to a non-physiological structural response
	Physiological verification of the coupled NMSK-FEA workflow	Availability of *in vitro* or *in vivo* reference data; measurable outputs; model scope; assumptions in load transfer	Verify the coupled workflow against physiological measurements where possible, such as joint kinematics, contact pressure, or other experimentally measurable responses, in addition to checking numerical consistency after load transfer	Verify the coupled workflow against physiological or experimental measurements where possible, such as structural response, strain, or other measurable outcomes, in addition to checking numerical consistency after load transfer

Although not derived directly from a single U.S. Food and Drug Administration (FDA) guideline, these recommendations are structured to align with broader FDA expectations for computational modelling and simulation, particularly with respect to context of use, transparent reporting of model assumptions and inputs, and credibility assessment through verification and validation (FDA, 2023).

### 3.1 Patient-specific geometry, meshing and material properties

Subject-specific FEA model development typically begins with selection of an appropriate imaging modality, followed by segmentation and geometry definition, surface preparation, volumetric meshing, material assignment, and model assembly (Taddei et al., 2007). In practical terms, x-ray computed tomography (CT) is generally better suited to bone-dominant models, particularly when clear cortical boundaries or density-based material mapping are required (Knowles et al., 2016). In contrast, MRI is typically preferred for cartilage, ligaments, menisci, and other soft tissues because of its superior soft-tissue contrast (Erdemir et al., 2019; Yan et al., 2024). When both accurate bony contours and soft-tissue geometries are needed, combined computed tomography (CT)–magnetic resonance imaging (MRI) workflows may offer complementary advantages, although they introduce additional registration challenges (Carey et al., 2014).

When representing soft tissues with composite structures, constitutive formulations must capture complex mechanical and structure-function relationships. For instance, the influence of collagen fibril networks in cartilage requires more complex material formulations, such as a fibril-reinforced poro-viscoelastic model. These structures have material properties informed by the literature and can range from simple elastic descriptions to more advanced viscoelastic or poro-viscoelastic models, depending on the tissue and the orthopaedic question being addressed. These modelling choices also influence numerical implementation: implicit solvers are commonly preferred for nonlinear quasi-static analyses and for advanced constitutive formulations, whereas explicit solvers may be advantageous in problems dominated by large deformations, contact discontinuities, or impact-type loading because of their favourable stability and computational efficiency in such settings.

For models developed directly from medical images, accurate segmentation alone is not sufficient. Reconstructed surfaces must be conditioned to support stable volumetric meshing and reliable contact analysis, particularly in orthopaedic applications involving contact interfaces (Gibbons et al., 2022; Burkhart et al., 2013). Smoothing is therefore necessary to reduce noise and staircasing artefacts, but should be applied conservatively as excessive smoothing may remove local anatomical features that meaningfully influence mechanical response (Burkhart et al., 2013; Cooper et al., 2025). This challenge is amplified in individuals with MSK disorders that cause irregular tissue remodelling, such as late-stage osteoarthritis with substantial cartilage loss and bone deformities. A practical guide is to smooth only the minimum amount required for numerical robustness while preserving local conformity in regions where contact or load transfer is important. One practical quality-control criterion is preservation of component volume during smoothing, indicating that the procedure does not alter the macro-scale geometry significantly. The consistency of subsequent material mapping, whereby CT-based Hounsfield units are converted to density and then to elastic material properties, can also serve as a useful quality-control step, as abrupt or non-physiological changes in assigned properties on the surface indicate geometric inaccuracies (Taddei et al., 2007). Mesh sensitivity should also be assessed for the primary outputs, with element type, mesh density, convergence criterion, and residual output change reported; a change below approximately 2% between successive refinements may be used as a practical criterion when justified for the application (Dreyer et al., 2024).

Full image-based generation of high-fidelity models can remain labour intensive. Consequently, the field is increasingly adopting faster personalisation strategies, including linear scaling of reference geometries, nonlinear morphing (Esrafilian et al., 2024), statistical shape modelling (Carman et al., 2022; Shi et al., 2022; Fernandez et al., 2023), and mesh-warping techniques that preserve mesh quality while adapting template anatomies to subject-specific shapes (Bavil et al., 2024). These approaches are particularly attractive for joint-level applications, where efficiency, robustness, reproducibility, and anatomical fidelity must be balanced.

### 3.2 Patient-specific boundary conditions

Patient-specific muscle and joint reaction forces predicted by NMSK models must be translated into loading and boundary conditions for FEA. The physiological credibility of FE predictions therefore depends, in part, on how faithfully the loading and boundary-condition environment is reproduced. However, while EMG-informed NMSK outputs provide the most physiologically specific loading inputs when reliable EMG data are available, the FE component of the coupled workflow is not intrinsically restricted to EMG-informed outputs. It can also be driven by muscle and joint loading estimates derived from non-EMG MSK approaches, provided that the assumptions, limitations, and expected uncertainty of those inputs are considered when interpreting outcomes. The translation of estimated joint and muscle forces primarily depends on the FE model assumptions and design, i.e., whether it represents a single organ or a multi-compartment system. For example, a single-organ FE model can represent whole bones whereas joint-level models are represented with multiple tissues (Karimi Dastgerdi et al., 2023).

In joint-level models, the objective is typically to produce boundary conditions that create realistic contact mechanics. The current evidence-informed recommendation is therefore to apply NMSK-estimated resultant joint forces and moments, often supplemented with (primary) kinematics, through a reference point representing the joint’s functional centre of rotation (or coordinate system origin within the NMSK model). This reference point is kinematically coupled to the relevant body, e.g., cartilage-bone interface nodes, to preserve correct force-moment relationships while avoiding artificial constraints on the articular tissues. In this setting, one side of the joint is commonly fixed and the other mobilised, such that joint kinematics together with joint contact forces and moments are prescribed in the coordinate system relative to the fixed body (Esrafilian et al., 2024). These joint moments may represent not only the net inverse-dynamics estimates, but also the contribution of muscle forces acting about the joint, either implicitly through superposition of moments derived from muscle forces onto the inverse-dynamics moments (Dastgerdi et al., 2025b), or explicitly by applying the forces of muscles spanning the joint along their respective lines of action (Esrafilian et al., 2021). Joint pose, contact state, and connecting tissue properties, such as ligament pre-tension, should also be considered part of the boundary condition setup, as they can substantially influence predicted tissue mechanics (Esrafilian et al., 2021).

In single-organ FE models (e.g., whole bones), a key challenge is constraining rigid body motion induced by muscle, joint, and other tissue forces while preserving their boundary effects on local deformations. These forces must first be expressed in the target bone coordinate system, while effective muscle lines of action should be derived from the attachment site and the next immediate via point, which together define the force transmitted to the bone surface at the attachment region in the NMSK model (Eghan-Acquah et al., 2025a). Once force vectors are derived, the next step is to couple anatomically relevant surface regions to reference points representing joint centres or muscle attachment locations in the NMSK model, thereby enabling consistent transfer of loads from the focal NMSK representation to regions on the FE model (Bavil et al., 2026; Bavil et al., 2025; Eghan-Acquah et al., 2024). A key consideration in FEA is the choice of boundary constraints applied to the whole bone. Insufficient constraining of the bone permits non-physiological rigid body motion, while excessive constraints can distort physiological relevance and predicted deformation of the model. Current recommendations therefore favour minimal, anatomically justified constraints (Eghan-Acquah et al., 2025a). In practical terms, this can be implemented using an isostatic 3-2-1 constraint strategy (Marin and Ferreira, 2000), in which three anatomically separated regions collectively remove the six rigid-body degrees of freedom without fully fixing a large bone surface. Constraint locations should reflect expected anatomical motion or directions of limited displacement, be applied through distributed coupling regions where possible, and be positioned to minimise artificial stress concentrations at the constrained regions. The selected constraint configuration should be checked through sensitivity analysis, preferably first in intact or representative bone models, to confirm that rigid-body motion is suppressed without introducing artificial reaction forces or non-physiological deformation. Inertia relief may also be considered for suitably balanced quasi-static loading cases where artificial fixation effects are difficult to avoid (Bavil et al., 2024; Speirs et al., 2007). While this issue has been examined in detail for the femur, comparable workflow considerations regarding boundary conditions remain underdeveloped for other single-bone FE models.

Consistent alignment of anatomical definitions and transfer of loading and boundary conditions across the NMSK and FE models is essential (Robinson et al., 2020; Tiew et al., 2025). This is particularly important when CT- or MRI-derived FE geometries are coupled with a linearly scaled or partially personalised MSK/NMSK model, because the two skeletal representations may not share identical morphology. Before load transfer, the image-derived FE geometry should be registered to the corresponding MSK/NMSK segment or joint coordinate system using consistent anatomical landmarks or coordinate-system definitions. Any mismatch in joint centres or axes, segment orientation, or muscle attachment should then be reconciled where possible or explicitly documented as a modelling assumption. An anatomically and mechanically consistent representation of tissues across both frameworks is required, particularly when muscles, tendons, or ligaments are represented. For instance, the global force-deformation or strain behaviour assumed in the NMSK model should be compatible with the local mechanical response of the corresponding FE representation to avoid cross-scale mismatch. A conventional approach to verify and adjust the NMSK-FEA modelling workflow is to model and reproduce *in vitro* or *in vivo* measurements, such as joint kinematics and contact pressure (Karimi Dastgerdi et al., 2023). Overall, current NMSK-FEA workflows still rely heavily on study-specific assumptions, highlighting the need for more standardised anatomy and load-transfer strategies between NMSK and FE models to improve reproducibility and broader clinical usability (Orozco et al., 2024).

### 3.3 FEA output measures

Output measures in FE analyses depend on the tissue modelled, the orthopaedic problem, and the underlying clinical or biological question; importantly, they need to be relevant and robust enough to support credible conclusions within model uncertainty (Huiskes and Hollister, 1993). For instance, in bone FE analyses, principal tensile and minimum principal (compressive) stresses or strains are most relevant for structural failure (Fleps et al., 2018; Dastgerdi et al., 2025c). Alternative failure criteria, such as the Coulomb-Mohr approach, have also been used in specific applications such as the radius (Edwards and Troy, 2012), whereas strain-based measures such as strain energy density are more relevant for mechanobiological processes including remodelling and stress shielding (Bavil et al., 2026; McNamara et al., 1997; Eghan-Acquah et al., 2025b). In long bones, additional descriptors such as cortical surface strain (Reikeras et al., 2011), whole-bone deflection (Papini et al., 2007), and apparent structural stiffness (Chevalier et al., 2007) provide useful indicators of load transfer and enable comparison with experimental or radiographic validation data.

In joint-level FE models, such as intact knee FE models with cartilage and menisci, the focus shifts to articular contact mechanics and soft-tissue loading (Dastgerdi et al., 2026). Accordingly, outputs that characterise the magnitude and distribution of contact interactions become central to interpretation. Contact pressure, contact area, and their spatial distribution are biologically relevant measures (Haut Donahue et al., 2002; Gu and Pandy, 2020). When advanced constitutive formulations are used, such as poro-viscoelastic or fibril-reinforced cartilage and menisci models, additional variables can be explored, including fluid pressure, fluid flow, and solid matrix stress (Esrafilian et al., 2021; Korhonen et al., 2006). These measures provide insight into mechanisms such as porous-viscous behaviour, collagen fibre tension, and matrix shear deformation, which are closely linked to the local mechanical environment experienced by chondrocytes and extracellular matrix components and may therefore relate more directly to processes of cartilage degeneration or damage (Mow et al., 1999). Modelling fluid flow within cartilage also relates to fluid exudation at the cartilage surfaces, which produces its characteristic low-friction behaviour (Esrafilian et al., 2021).

For metallic orthopaedic implants, various mechanical variables have importance. Von Mises stress is an appropriate primary indicator of structural demand, especially when fatigue governs the intrinsic structural durability of an implant, particularly in load-bearing components (Eberle et al., 2010). In articulating components, contact variables such as contact stresses, pressure distribution, sliding distance and multidirectional motion are relevant to wear behaviour (Wang et al., 2013). Finally, implant performance is governed by fixation stability and the transfer of mechanical loads to surrounding tissues, particularly bone, where localised or low strain energy densities are key determinants of bone atrophy and implant loosening (Eghan-Acquah et al., 2025a; McNamara et al., 1997).

The bone-implant interface and interfaces between bone fragments are clinically meaningful. Bone-implant micromotion, for example, is a key indicator of primary fixation stability and likely osseointegration. Bone interfragmentary movement in fracture or osteotomy models reflects construct stability and the mechanical environment for healing (Bavil et al., 2026; Bavil et al., 2025). When interfragmentary healing is modelled explicitly, callus differentiation can also be estimated using mechanoregulatory stimuli such as deviatoric strain and related fluid-pressure measures (Bavil et al., 2026). Interface measures therefore frequently provide more clinically actionable insight than stress distributions within individual components alone.

Regardless of the specific variables selected, FE outputs should always be interpreted considering constitutive assumptions, modelling simplifications, and available validation evidence. Stress outputs near contact interfaces should be interpreted cautiously, as local singularities or artificial stress concentrations may substantially overpredict the mechanical response. Elements with stresses more than 10% higher than neighbouring elements can be treated as potential stress singularities and investigated while persistent occurrences may also indicate meshing or contact-definition problems (Bavil et al., 2025). Additionally, variables that are poorly linked to the biological response of interest can lead to misleading conclusions even when the numerical solution itself is robust (see Table 2).

## 4 Case studies

The following case studies illustrate how coupled NMSK-FEA workflows can be applied to evaluate the patient-specific mechanical consequences of candidate surgical strategies in joint-level and single-organ modelling contexts. They are intended to demonstrate workflow implementation and the potential value of these approaches for informing personalised biomechanical assessment and surgical decision-making. However, these predictions should be interpreted as conditional assessments of the immediate postoperative mechanical environment, rather than forecasts of longer-term postoperative adaptations.

### 4.1 Case study 1. Anterior cruciate ligament reconstruction

In anterior cruciate ligament reconstruction (ACLR), the clinical question is no longer simply whether the graft restores passive stability, but which patient-specific surgical configuration is most likely to restore favourable postoperative knee mechanics during functional loading. In this context, the research question was whether a coupled NMSK-FEA workflow could be used as a preoperative decision-support tool to compare candidate ACLR strategies and identify the configuration that best reproduces subject-specific joint mechanics. This framing follows the broader premise: the mechanically optimal solution is not defined by surgical parameters in isolation, but by how those parameters interact with anatomy and dynamic loading. Indeed, recent ACLR studies have shown that predicted outcomes depend on the interaction between surgical parameters, anatomical variation, and functional loading, with substantial inter-subject variability in joint kinematics and cartilage stresses even for nominally similar reconstructions (Dastgerdi et al., 2025b; Díaz-Cuadro et al., 2022; Naghibi et al., 2020; Risvas et al., 2022).

To evaluate the mechanical effects of different ACLR surgical scenarios, we designed the case study according to the joint-level recommendations in Table 2. Subject-specific MRI was used to define the joint geometry, while the EMG-informed NMSK model provided the subject-specific loading environment during the stance phase of the gait. The FE model was constructed to reproduce articular contact mechanics, and the primary transferred inputs were joint contact forces, joint moments, muscle forces superimposed on knee moments, and time-varying joint kinematics. These quantities were applied using anatomically consistent definitions and coordinate systems, with loading and kinematics represented relative to the fixed body of the joint, the tibia, so that the force-moment relationship was preserved. The initial joint pose, contact state, and ligament behaviour were also treated as parts of the boundary-condition definition to align the joint state with the first frame of the NMSK loads. Joint kinematics and stress distributions on the contact surfaces were selected as outputs because they were clinically relevant to the risk of post-traumatic osteoarthritis in the reconstructed knee.

**FIGURE 1:**
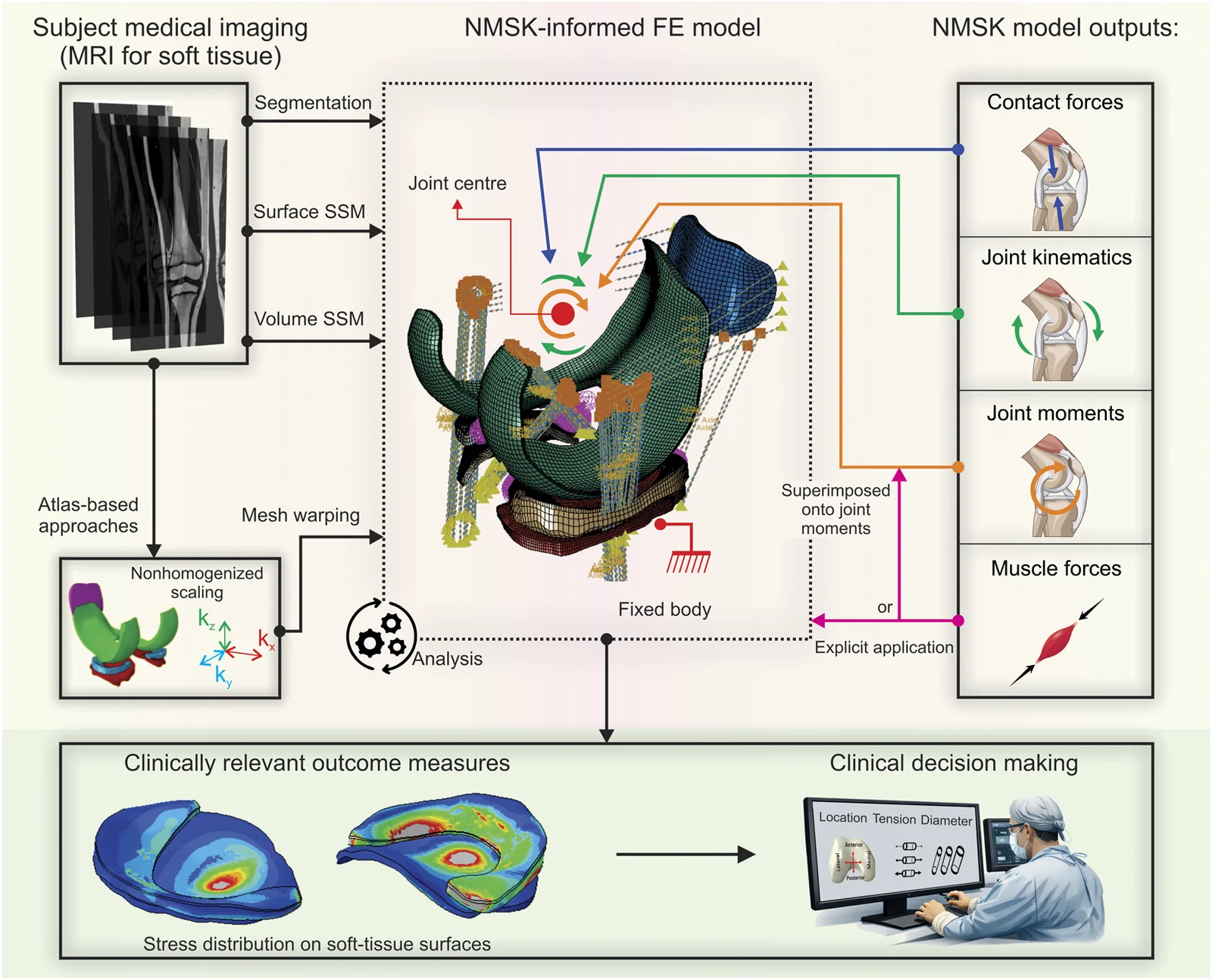
Workflow for generating an EMG-informed NMSK-FEA model of the tibiofemoral joint. Subject-specific MRI data were used to derive the geometry of the distal femur, proximal tibia, patella, menisci, and cruciate and collateral ligaments. Contact forces, joint kinematics, joint moments, and muscle forces from NMSK were transferred as subject-specific boundary and loading conditions. The primary outcome measures were the stress distributions on the joint contact surfaces. This digital twin workflow can be used as a decision support tool for preoperative planning of ACLR surgeries. MRI: Magnetic Resonance Imaging, NMSK: Neuromusculoskeletal, FE: Finite Element, SSM: Statistical Shape Modelling.

Scientifically, this case study showed that the mechanical consequences of ACLR depended on the interaction between tunnel placement, graft pretension, graft type, and graft diameter under subject-specific loading conditions, rather than on any single surgical variable in isolation (see Figure 1). Clinically, the coupled framework enabled five tunnel placements, three pretension levels, three graft types, and three graft diameters to be evaluated for each patient, with the optimal configuration defined as the one that produced the smallest deviation from the contralateral knee in joint kinematics and cartilage stress. In this way, the model informed preoperative decision-making by identifying patient-specific graft and tunnel configurations that best balanced restoration of normal joint motion with physiological cartilage loading.

### 4.2 Case study 2. Proximal femoral osteotomy

In proximal femoral osteotomy (PFO), the clinical question is which patient-specific correction strategy is most likely to create a favourable mechanical environment for fixation stability and bone healing. Our research question was therefore whether a coupled NMSK-FEA workflow could be used to compare candidate postoperative alignments and identify the correction that best optimises load transfer within the femur-implant construct. This reflects the broader premise of this paper that the mechanically optimal surgical solution is not universal but depends on how alignment changes interact with subject-specific anatomy and functional loading.

**FIGURE 2:**
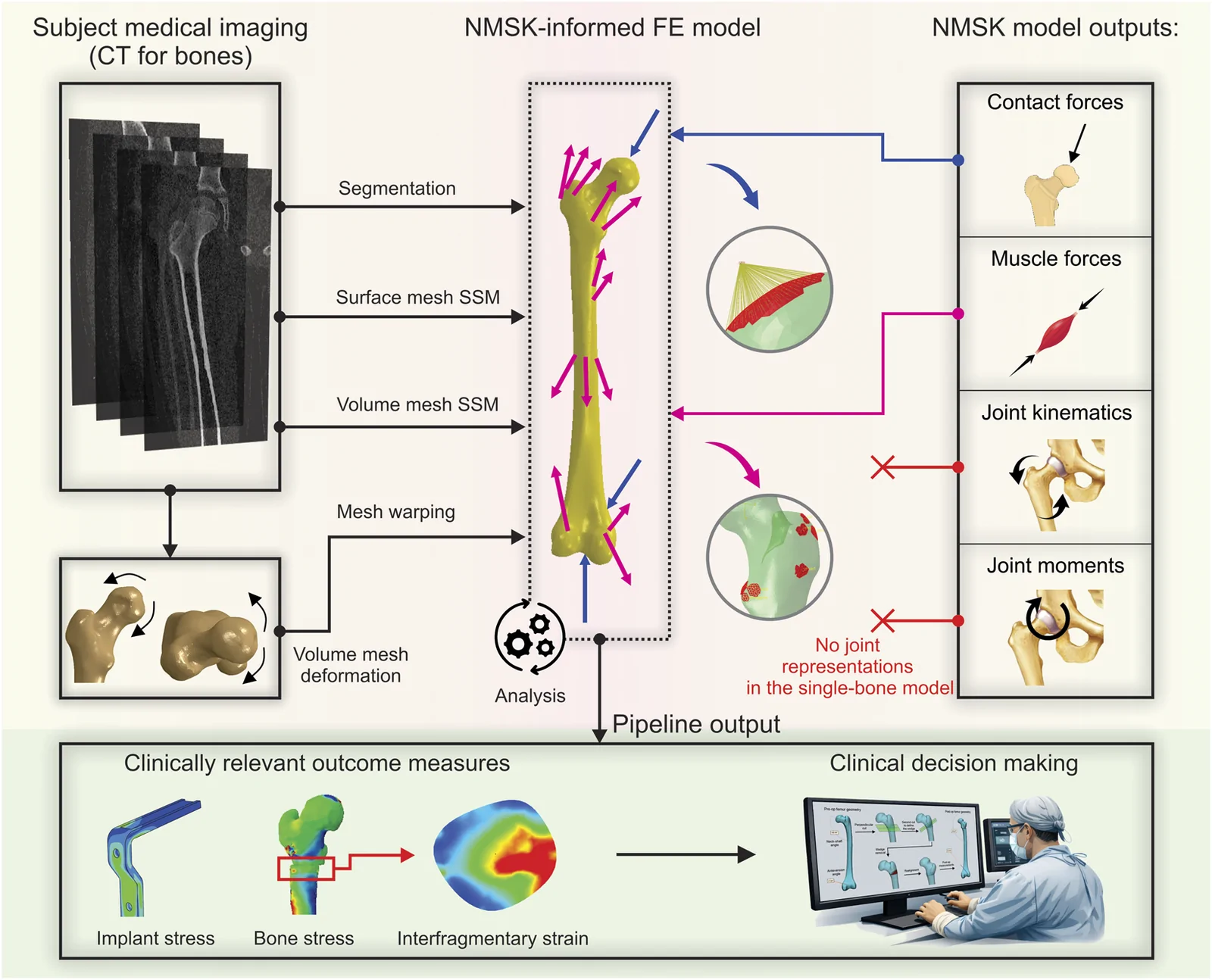
Workflow for generating an EMG-informed NMSK-FEA model for femur or femur-implant constructs. Subject-specific CT data were used to derive the geometry of the bone. Muscle and joint contact forces from NMSK were transferred as subject-specific boundary and loading conditions. The primary outcome measures were the maximum principal stresses on the bone, von Mises stresses on implants, and interfragmentary mechanics. This digital twin workflow can be used as a decision support tool for preoperative planning of osteotomies. CT: Computed Tomography, NMSK: Neuromusculoskeletal, FE: Finite Element, SSM: Statistical Shape Modelling.

The case study was designed according to the single-bone recommendations in Table 2. Subject-specific CT data were used to generate the femoral geometry, and the FE model was constructed as an organ-level representation of the orthopaedic system. Accordingly, the EMG-informed NMSK outputs transferred to the FE model were muscle and joint contact forces, while joint kinematics and joint moments were not directly imposed because the model did not include a joint representation. Consistent with the recommendations, muscle lines of action were derived from the anatomical definitions in the NMSK model, loads were transformed into the femoral coordinate system, and forces were applied through anatomically relevant coupling regions to preserve physiological load transfer. The primary outputs were selected to match the clinical question, including bone-implant micromotion, implant stress, and the broader mechanical environment associated with healing (see Figure 2).

A key practical challenge in this case study was the lack of standardised boundary-condition strategies for single-bone loading scenarios. Preventing rigid body motion without over-constraining the femur is especially difficult in these models, and this issue was recently addressed systematically by Bavil et al. (2024), which helped formalise the minimal, anatomically justified constraint strategy highlighted in Table 2. The case study showed that postoperative mechanics were highly patient-specific. Variation in neck-shaft angle and anteversion angle altered the bone-implant micromotion and implant stress differently across patients, and the same nominal correction could produce markedly different mechanical responses. More recent analyses further showed that variation in postoperative neck-shaft angle could also alter interfragmentary mechanics and, in some femurs, shift the predicted healing mode. Clinically, this workflow was used to address several surgical planning questions, most notably which postoperative neck-shaft angle after corrective osteotomy was most likely to promote direct healing while reducing failure risk for the specific anatomy and loading conditions of each patient. These findings indicate that preoperative planning should not rely on a universal postoperative neck-shaft angle but instead use patient-specific modelling to identify the correction that best balances fixation stability, implant loading, and the local mechanical environment for healing.

## 5 Uncertainty, error propagation, and model credibility

Coupled NMSK-FEA workflows integrate multiple experimental, modelling, and numerical stages, and uncertainty can enter at each stage. In the NMSK component, relevant sources include marker placement, soft-tissue artefact, ground reaction force assignment, EMG electrode placement and processing, model scaling, joint definition, muscle-tendon parameter calibration, and assumptions used to estimate unmeasured muscle excitations. In the FE component, uncertainty may arise from image resolution, segmentation, geometry smoothing, mesh density and quality, material assignment, coordinate-system registration, load transfer, and boundary conditions. These sources do not necessarily influence the final predictions independently; errors in earlier stages can propagate into downstream loading conditions and alter the predicted mechanical response of joint-level or single-organ FE models. The importance of each uncertainty source depends on the intended use of the workflow and the output measures used to support interpretation. For joint-level models, the dominant uncertainty sources depend on the research question and output measure: transferred kinematics and loading, model complexity, ligament constitutive representation, and patient-specific geometry may each influence predicted mechanics differently across applications (Bolcos et al., 2018). For single-organ models, uncertainty in muscle and joint force directions, attachment region definition, material mapping, and constraint strategy may strongly affect predicted stress, strain, or structural stiffness. Therefore, uncertainty assessment should be designed around the clinical or biomechanical question, model type, and output measures of interest.

A practical credibility strategy is to identify the dominant uncertainty sources and influential parameters for the intended application, verify each workflow step, and perform targeted sensitivity analyses for inputs expected to influence the selected outputs. This includes conventional checks such as reporting model calibration and moment-tracking errors, confirming force and moment equilibrium after load transfer, performing mesh convergence or mesh sensitivity analyses, evaluating sensitivity to material properties and contact parameters, and comparing outputs with *in vivo*, *in vitro*, radiographic, or literature-based reference data where available. It also includes application-specific sensitivity analyses that directly test how candidate clinical or anatomical variables influence the outputs used for decision support. In the ACLR case study, for example, sensitivity analysis was used to determine which reconstruction parameters had the greatest influence on FE-predicted knee mechanics (Dastgerdi et al., 2024). In the PFO case study, sensitivity analyses examined how key femoral anatomical parameters, particularly neck-shaft angle and anteversion angle, affected postoperative mechanical outcomes (Bavil et al., 2025).

These examples illustrate that alternative surgical strategies can be virtually trialled under patient-specific loading conditions, and that uncertainty and sensitivity analyses should not only focus on technical modelling parameters, but also on the clinical variables that may drive treatment selection. When alternative surgical strategies are compared across multiple subjects, inter-subject variability should also be quantified by reporting individual responses alongside cohort-level ranges, distributions, or probabilistic summaries where sufficient data are available. Deterministic sensitivity analyses may be appropriate for identifying dominant surgical or anatomical parameters in small patient-specific cohorts, whereas probabilistic approaches, Monte Carlo sampling, virtual cohorts, or population-level statistical analyses may be required to quantify uncertainty and variability across broader patient groups. Identifying dominant parameters and inter-subject variability is important before routine clinical decision-making, because model-informed recommendations should distinguish robust treatment effects from outputs that are highly sensitive to patient anatomy, loading, surgical parameters, or modelling assumptions. When full quantitative uncertainty propagation or full anatomical personalisation is not feasible, assumptions and modelling limitations should be reported explicitly. In such cases, simplified models may still be useful for comparative analyses between candidate interventions, provided that outputs are interpreted as relative mechanical trends rather than fully personalised absolute predictions. Model outputs should therefore be presented as decision-support evidence within their credibility limits rather than as deterministic clinical recommendations.

## 6 Overview and future direction

**FIGURE 3:**
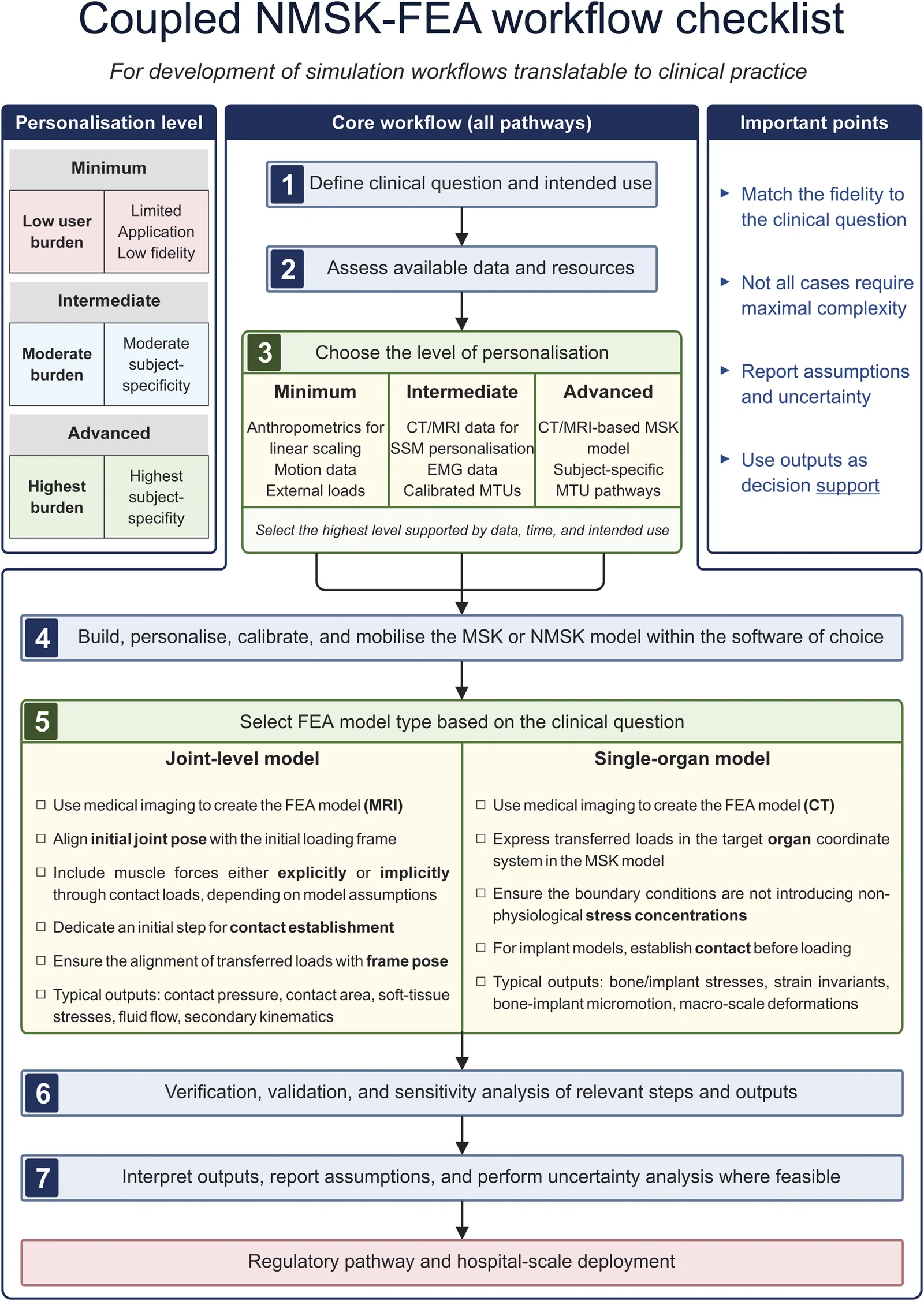
Coupled NMSK-FEA workflow checklist for developing simulation workflows translatable to clinical practice. The checklist summarises the core steps common to coupled NMSK-FEA workflows. The figure highlights key decision points where the workflow may diverge according to available resources, intended use, and model type. NMSK, neuromusculoskeletal; FEA, finite element analysis; MSK, musculoskeletal; CT, computed tomography; MRI, magnetic resonance imaging; EMG, electromyography; SSM, statistical shape modelling; MTU, muscle-tendon unit.

The core methodological components required for patient-specific orthopaedic biomechanics already exist and have been demonstrated across multiple applications. Advances in motion capture, wearable sensors, medical imaging, automated model personalisation, EMG-informed NMSK modelling, and computational efficiency now make it possible to generate subject-specific anatomical models and loading conditions within clinically meaningful timeframes. The key challenge is no longer only technical development, but translation into tools that can support real clinical decisions. Hospitals are often cautious in adopting new technologies when outputs do not map clearly onto specific actions, making it uncertain how results should inform treatment planning, intervention selection, or implant choice, or whether their use will translate into safer, more effective, and more cost-effective care. This problem is amplified when uncertainty is reported without practical clinical interpretation, and by unresolved questions around the boundaries between model output and clinician judgement, including liability and accountability when these tools influence care. For clinical adoption, outputs must therefore be presented in surgeon-facing terms, such as recommended graft tension, tunnel position, correction angle, implant position, fixation strategy, or predicted trade-offs between treatment options, rather than as raw mechanical variables. In this context, the value of coupled NMSK-FEA lies in augmenting surgeon decision-making by converting detailed mechanics into objectively informed surgical options (see Figure 3 for an overview of the presented recommendations).

Translation of NMSK-FEA frameworks into clinical workflows will require standardised methods for transferring NMSK outputs to FE boundary conditions, consistent anatomical definitions across modelling platforms, and reporting of clinically relevant output measures. Equally important is informed reporting that makes model assumptions explicit and addresses data uncertainty, sources of error, and their propagation through the modelling workflow. Translation also requires workflows to be designed with regulatory and implementation requirements in mind, rather than methodological validity alone. Intended use must be clearly defined, validation must address clinical decision impact as well as model accuracy, and prospective clinical studies are needed to determine whether model-informed planning improves surgical decisions, functional or radiographic outcomes, revision risk, or cost-effectiveness compared with current standard planning approaches. Such studies should also capture postoperative movement adaptations, so that future workflows can distinguish immediate mechanically favourable surgical configurations from those that remain favourable under longer-term functional loading. Longitudinal, multi-centre datasets linking preoperative movement, imaging, surgical decisions, postoperative adaptation, and outcomes will be important for developing future hybrid mechanistic and data-driven workflows that can evaluate not only immediate mechanical consequences, but also longer-term adaptation patterns. Development must account for medical device and software-as-a-medical-device requirements.

From an implementation perspective, established expert-led pipelines such as those presented in the case studies are already compatible with many elective preoperative planning timelines once the required data are available, with full patient-specific NMSK-FEA analyses typically requiring several working days to approximately 1 week. However, routine point-of-care implementation will require further automation of analysis steps prior to surgeon-facing reporting. Accordingly, the main remaining barriers are scalability, standardisation, and clinical integration. These barriers include manually intensive data preparation, limited interoperability with hospital information systems, and reliance on research-grade software outside its licensed scope. Collectively, these issues can disrupt clinical workflows and add cognitive and time burdens that hinder adoption despite strong technical promise. With continued improvements in workflow automation and rapid motion analysis, coupled NMSK-FEA workflows are increasingly capable of supporting clinically relevant “what-if” evaluations of surgical strategies and implant configurations before intervention, enabling orthopaedic decision-making to move beyond population averages towards patient-specific mechanical assessment at point of care. Promising opportunities for extension also exist in amputee care, where coupled NMSK-FEA workflows could build on established technologies for osseointegrated prostheses, including rehabilitation load monitoring and assessment of loading reliability and compliance, to support patient-specific implant and rehabilitation decision-making (Frossard et al., 2010; Vertriest et al., 2017; Vertriest et al., 2015). Without standardisation, automation, clinical integration, and appropriate regulatory pathways, even technically robust workflows are likely to remain confined to bespoke research implementations rather than routine clinical practice.

## Data Availability

The raw data supporting the conclusions of this article will be made available by the authors, without undue reservation.
